# Cerebellar-limbic dysregulation and sensorimotor network alterations in male primary insomnia: a resting-state functional magnetic resonance imaging study

**DOI:** 10.3389/fnhum.2025.1633506

**Published:** 2025-09-02

**Authors:** Juan Lu, Zhengzhen Yuan, Jidan Hu, Jiajun Yue, Pingping Jie, Yong Liu, Haiyi Zhang, Jie Zhao

**Affiliations:** ^1^Department of Magnetic Resonance Imaging, The Affiliated Traditional Chinese Medicine Hospital, Southwest Medical University, Luzhou, China; ^2^School of Physical Education, Southwest Medical University, Luzhou, China; ^3^Department of Radiology, The Second People’s Hospital of Neijiang, Neijiang, China

**Keywords:** insomnia disorder, male, resting state functional magnetic resonance imaging, ALFF, ReHo

## Abstract

**Objective:**

The neuropathological mechanisms specific to male insomnia disorder (ID) remain underexplored, particularly regarding intrinsic brain activity patterns. Using functional magnetic resonance imaging (fMRI), this study aims to investigate the characteristics of amplitudes of low-frequency fluctuations (ALFF) and regional homogeneity (ReHo) in male patients with primary ID, and to explore the correlations between these neuroimaging indicators and sleep scale scores.

**Methods:**

A total of 30 male patients diagnosed with ID and 30 age-matched healthy controls (HCs) were enrolled. All participants underwent standardized assessments with the Insomnia Severity Index (ISI), Pittsburgh Sleep Quality Index (PSQI), and Hamilton Depression Scale (HAMD). Resting-state functional magnetic resonance imaging (rs-fMRI) was utilized to assess regional brain activity abnormalities in male insomnia patients through amplitude of low-frequency fluctuations (ALFF) and regional homogeneity (ReHo) analyses. Statistical correlations between aberrant ALFF/ReHo values and clinical scale scores were subsequently examined.

**Results:**

Patients with ID showed significantly higher scores on the PSQI, HAMD Rating Scale score, and the ISI, compared with HCs. fMRI results revealed that, relative to HCs, ID patients exhibited reduced ALFF in the Precentral_R and increased ALFF in the Cerebelum_6_R and Temporal_Inf_L (*p* < 0.05, cluster level-FWE corrected). Regarding ReHo, patients displayed elevated ReHo values in the Temporal_Inf_L, Cerebelum_6_R, and Hippocampus_R, whereas decreased ReHo values were observed in the Putamen_L, Insula_R, and Calcarine_R (*p* < 0.05, cluster level-FWE corrected). However, neither ALFF nor ReHo measures demonstrated significant correlations with clinical scale scores (*p* > 0.05).

**Conclusion:**

Male patients with ID exhibit functional abnormalities in the cerebellum-limbic system circuit and the sensorimotor network. These alterations are accompanied by impaired motor coordination, dysregulated emotional processing, and deficits in sensory integration. Although ALFF and ReHo metrics show no significant correlation with the severity of clinical symptoms, the regional distribution patterns of these indicators across different brain areas suggest that they may serve as an index for ID, rather than a direct reflection of symptomatic manifestations.

## 1 Introduction

Insomnia disorder (ID) is a mental health condition characterized by persistent difficulties in initiating or maintaining sleep, leading to significant daytime impairments such as cognitive deficits and mood disturbances (Chellappa and Aeschbach, 2022). It is also associated with an increased risk of depression and suicidal behavior. The WHO reports that 27% of the global population experiences chronic insomnia (Sutton, 2021). However, fewer than half of these cases receive standardized diagnosis or treatment (Li S. et al., 2016), highlighting the limited understanding of its underlying mechanisms. Resting-state functional magnetic resonance imaging (rs-fMRI), which measures blood oxygen level-dependent (BOLD) signals, provides a non-invasive tool to investigate dynamic functional brain networks (Ogawa et al., 1990) and offers new insights into the neural underpinnings of ID.

Accumulated evidence suggests that disrupted functional connectivity within core neurocognitive networks, including the default mode network (DMN), the salience network (SN), and the central executive network (CEN), underlies ID (Santarnecchi et al., 2018). A hallmark finding is hyperactivity in the DMN, which may reflect impaired wake/sleep homeostasis and maladaptive cognitive-emotional processing. Despite progress, critical gaps persist in mapping sex-specific neural substrates of ID, particularly in males (Mallampalli and Carter, 2014), as the behaviors associated with sleep disturbances in this population remain systematically underexplored.

Epidemiological data reveal a striking 1.5- to 2-fold female predominance in the prevalence of ID (Suh et al., 2018). Notably, while ID patients of both sexes exhibit similar clinical manifestations and share comparable hyperarousal mechanisms (Dai et al., 2016), they show significant sex-based differences in regional brain activity abnormalities. Compared with females, males exhibit reduced pain sensitivity linked to sleep disorders (Olia et al., 2025), which suggests sex-specific pathophysiological divergences in insomnia. This clinical dichotomy highlights the need to delineate male-specific mechanistic pathways. Current neuroimaging paradigms predominantly focus on mixed-sex cohorts or female-predominant samples, a methodological limitation that obscures male-specific circuit-level signatures.

In light of this background, our study employed rs-fMRI to compare functional neural profiles between male ID patients and HCs. Our primary objectives were twofold, firstly, to identify male-specific brain functional alterations and their underlying patterns in insomnia, secondly, to evaluate multimodal correlations between amplitude-based metrics, specifically the amplitude of low-frequency fluctuations (ALFF), regional homogeneity (ReHo), and clinically validated measures, including the Insomnia Severity Index (ISI), Pittsburgh Sleep Quality Index (PSQI), Hamilton Depression Scale (HAMD), insomnia severity scores, and insomnia-related cognitive dysfunction. These findings will contribute to the development of tailored diagnostic approaches and personalized treatment strategies for male patients with insomnia.

## 2 Materials and methods

### 2.1 Participants

A total of 30 male ID patients and 30 healthy controls (HCs) were enrolled in this study. Participants were recruited from the outpatient department of the Traditional Chinese Medicine Hospital Affiliated with Southwest Medical University between 2023 and 2024. All patients were diagnosed with ID according to the DSM-5 (Diagnostic and Statistical Manual of Mental Disorders, Fifth Edition). One hour before MRI scanning, all participants completed the ISI, PSQI, and HAMD assessments. Inclusion criteria for patients were (1) aged 25–60 years, (2) ISI ≥ 8, PSQI ≥ 8, and HAMD ≤ 8, and (3) Right-handedness. Exclusion criteria included (1) abnormal signals confirmed by routine cranial MRI, (2) insomnia secondary to substance use, medications, stimulants, or comorbid psychiatric/systemic diseases, and (3) contraindications for MRI.

The normal control participants were from family members, colleagues, and hospital staff, and the two groups were relatively matched for sex, age, and education. Inclusion criteria were: (1) age 25–60 years old, male, (2) All subjects demonstrated satisfactory sleep quality and were free of clinically diagnosed neurological conditions for at least 30 days before enrollment, (3) Right-handedness, (4) No MRI contraindications and normal findings on conventional MRI, (5) PSQI < 8, HAMD < 8, ISI < 8, (6) Participants were instructed to abstain from sleep deprivation, excessive alcohol consumption, and stimulant intake (including caffeinated beverages such as tea and coffee) for at least 48 h before MRI scanning.

All participants provided written informed consent following the Declaration of Helsinki. The study protocol was approved by the Ethics Committee of the Traditional Chinese Medicine Hospital at Southwest Medical University.

### 2.2 fMRI scanning

Imaging data were acquired using a Siemens Skyra 3.0 T MRI scanner (Siemens Healthineers) equipped with a 16-channel head–neck coil. Before rs-fMRI acquisition, structural scans including axial T1-weighted imaging (T1WI), T2-weighted imaging (T2WI), and T2-fluid-attenuated inversion recovery (T2-FLAIR) were performed to exclude participants with intracranial abnormalities.

During the resting-state scan, the participants were instructed to remain supine, breathe quietly with eyes closed, and minimize any mental activity. A foam pad was used to stabilize the head and reduce motion artifacts, and earplugs were provided to attenuate environmental noise. The scanning commenced after the participant acclimated to the environment. RS-fMRI data were acquired using a gradient-echo echo-planar imaging (EPI) sequence sensitive to BOLD contrast. Scan parameters included: repetition time (TR) = 2,720 ms, echo time (TE) = 40 ms, flip angle = 90°, slice thickness = 4.00 mm, field of view (FOV) = 240 mm × 240 mm, matrix size = 64 × 64, number of slices = 38, and a total of 270 time points were collected. Additionally, high-resolution T1-weighted structural images were obtained using a three-dimensional magnetization-prepared rapid gradient-echo (MPRAGE) sequence. Parameters for the MPRAGE sequence were as follows: TR = 1960 ms, TE = 2.98 ms, slice thickness = 1.00 mm, FOV = 256 mm × 256 mm, matrix size = 256 × 256, flip angle = 15°, and the number of sagittal slices was 176.

### 2.3 Data processing

#### 2.3.1 Preprocessing

Based on the MATLAB R2013b platform and utilizing the Restplus software, the following preprocessing steps were performed: (1) The data from the first 10 time points were excluded, and fMRI data from the subsequent 260 time points were included in the analysis, (2) Slice timing correction was applied to adjust for temporal differences across slices, (3) Head motion correction was conducted, with subject images exhibiting head motion translation exceeding 2.5 mm or rotation movement exceeding 2.5 degrees being excluded, (4) Spatial normalization was performed by registering BOLD images to their corresponding T1 structural images, followed by transformation to the standard Montreal Neurological Institute (MNI) space template.

#### 2.3.2 ALFF and ReHo analysis

We conducted a comprehensive analysis of ALFF and ReHo data based on the methodology proposed by Rocca et al. (2023) and Wang et al. (2016), ultimately obtaining standardized zALFF and szKccReHo for subsequent statistical analyses.

#### 2.3.3 Statistical analysis

Basic subject data were analyzed using SPSS 26.0 statistical software. Age data followed a normal distribution, and two-sample *t*-test was employed. The remaining variables (years of education, PSQI, ISI, and HAMD-24 scale scores) were assessed using the Mann–Whitney *U* test. Preprocessed fMRI data were corrected for multiple comparisons using Family-Wise Error (FWE) correction in SPM 12.0 software. Brain regions with corrected *p* < 0.05 were considered statistically significant. For the statistical analyses of ALFF and ReHo measures, we employed rigorous multiple comparisons correction using the family-wise error (FWE) method with a two-level thresholding approach, voxel-level threshold of *p* < 0.001 for initial screening of significant clusters, followed by a cluster-level threshold of *p <* 0.05 for final determination of statistically significant regions. To ensure the specificity of our findings, we systematically controlled for potential confounding effects of demographic variables by including both age and education level as covariates in all group-level analyses. MNI coordinates were selected, and brain regions were reported using peak coordinates derived from the cluster-level analysis. Pearson correlation analysis was performed to examine the relationships between these brain regions and PSQI, ISI, and HAMD-24 scale scores.

## 3 Results

### 3.1 Clinical data

**Figure 1:**
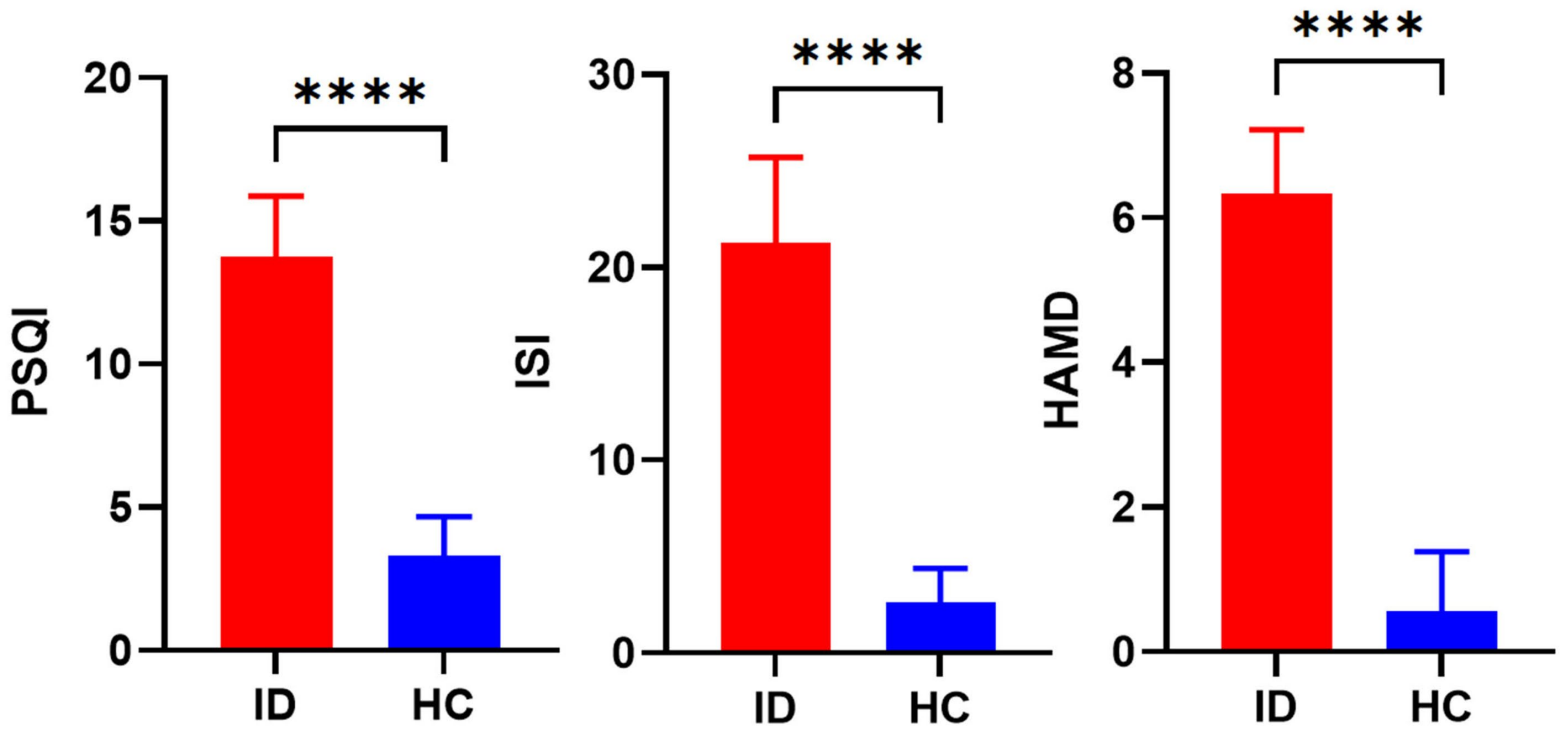
The bar chart shows that there are significant statistical differences in HAMD, PSQI, and ISI values between the ID group and the HC group. ****(*p* < 0.0001).

This study included 30 male patients with insomnia and 30 healthy male controls. There were no statistically significant differences in age (*p* > 0.05) or education level (*p* > 0.05) between the primary insomnia group and the control group (Table 1). However, the PSQI, HAMD, and ISI scores of the insomnia group were significantly higher than those of the control group, with statistically significant differences (*p* < 0.001) (Figure 1).

**Table 1:** Comparison of the clinical data.

	Insomnia group	Control group	*T/Z*	*p*
Age (years)	43.67 ± 2.03	43.26 ± 2.53	0.125	0.901
Educational level (years)	10.10 ± 0.95	10.11 ± 1.11	−0.055	0.956
PSQI	13.14 ± 0.70	3.74 ± 0.29	−4.988	<0.001
ISI	20.00 ± 1.40	2.32 ± 0.38	−4.922	<0.001
HAMD-24	6.10 ± 0.21	2.32 ± 0.38	−4.986	<0.001

ISI, Insomnia Severity Index; PSQI, Pittsburgh Sleep Quality Index; HAMD, Hamilton Depression Scale, unless otherwise stated, data are mean ± standard deviation.

### 3.2 Change in ALFF values

**Figure 2:**
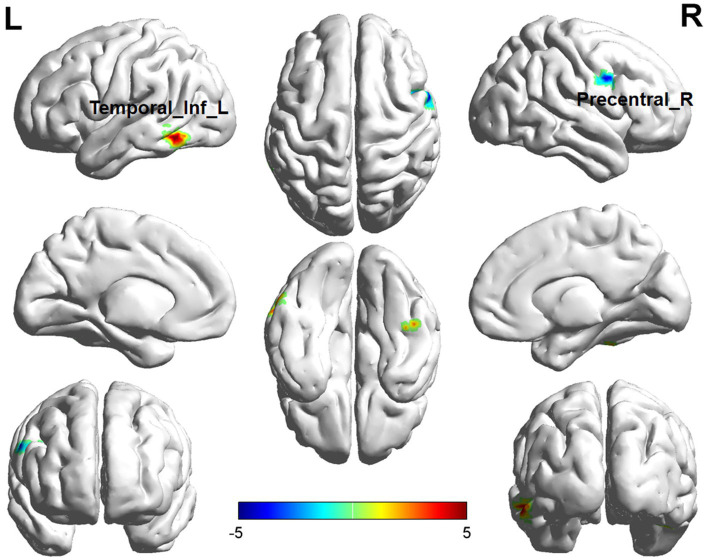
Brain regions (Temporal_Inf_L, Precentral_R) showing significant ALFF in the ID group compared with the HC group on the 3D template (*p* < 0.05, cluster level-FWE corrected). The color scale represents the *T*-value. Cool colors (blue) indicate a significant decrease in value, and warm colors (red) indicate a significant increase in value. R = right, L = left.

**Figure 3:**
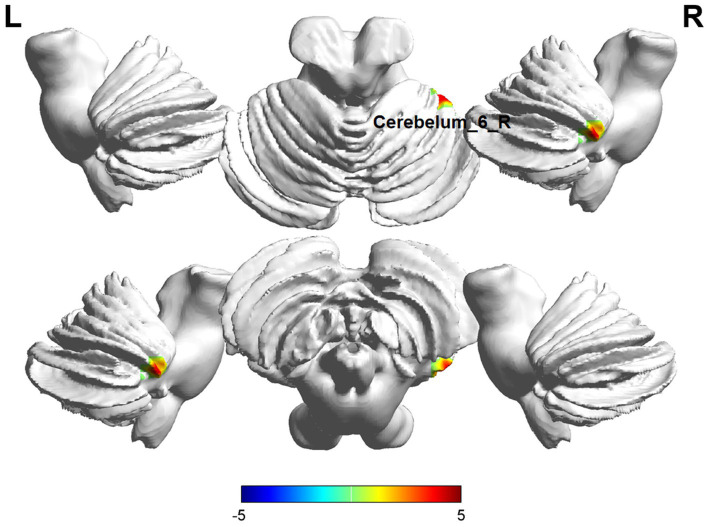
Brain regions (Cerebelum_6_R) showing significant ALFF in the ID group compared with the HC group on the 3D template (*p*  < 0.05, cluster level-FWE corrected). The color scale represents the *T*-value. Cool colors (blue) indicate a significant decrease in value, and warm colors (red) indicate a significant increase in value. R = right, L = left.

Comparisons between the insomnia and control groups revealed 3 brain regions showing significant between-group differences (*p* < 0.05, cluster level-FWE corrected, Table 2). The increased ALFF value was observed in the Cerebelum_6_R and Temporal_Inf_L, while decreased ALFF values were observed in the Precentral_R (Figures 2, 3).

**Table 2:** Brain region differences between HC and ID based on ALFF.

Brain regions^a^	Voxel (mm^3^)	AAL	MNI coordinates			*T* value
			*x*	*y*	*z*	
HC < ID						
Cluster 1	39		39	−36	−36	4.5222
Cerebelum_6_R	26					
Cluster 2	47		−57	−54	−15	5.6217
Temporal_Inf_L	37					
HC > ID						
Cluster 1	38		51	9	30	−4.7594
Precentral_R	33					

^a^Peak point of the selected brain region cluster.

### 3.3 The ReHo values were changed

**Figure 4:**
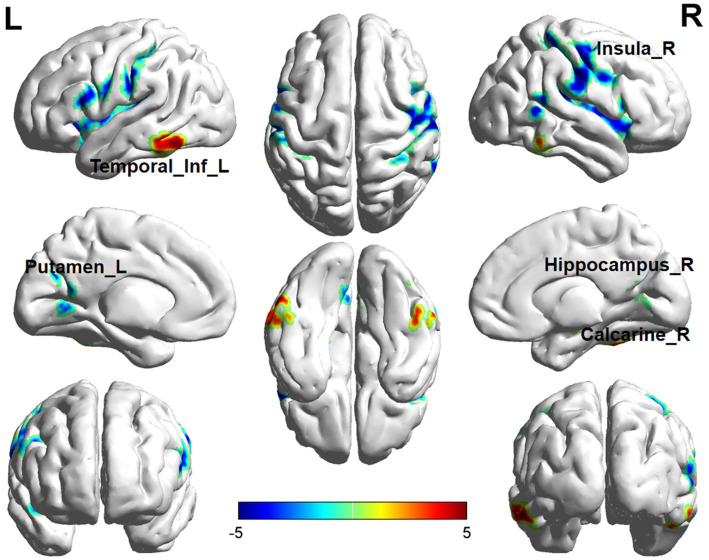
Brain regions (Temporal_Inf_L, Hippocampus_R, Putamen_L, Insula_R, Calcarine_R) showing significant ReHo in the ID group compared with the HC group on the 3D template (*p <* 0.05, cluster level-FWE corrected). The color scale represents the *T*-value. Cool colors (blue) indicate a significant decrease in value, and warm colors (red) indicate a significant increase in value. R = right, L = left.

**Figure 5:**
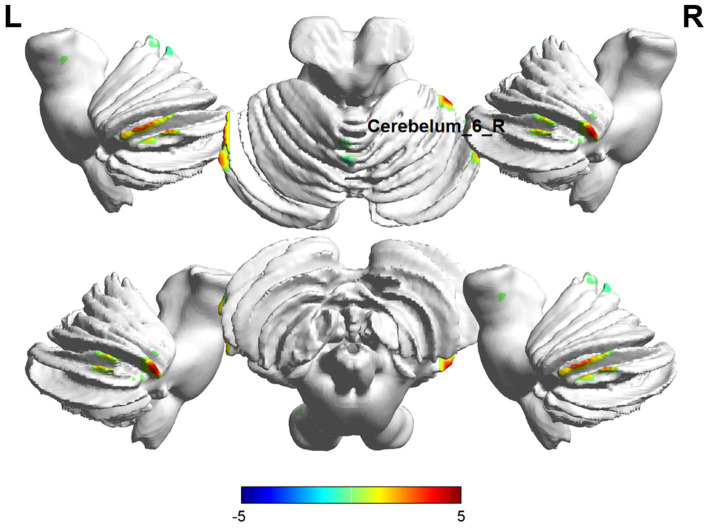
Brain regions (Cerebelum_6_R) showing significant ReHo in the ID group compared with the HC group on the 3D template (*p* <  0.05, cluster level-FWE corrected). The color scale represents the *T*-value. Cool colors (blue) indicate a significant decrease in value, and warm colors (red) indicate a significant increase in value. R = right, L = left.

In ReHo value comparisons, six brain regions demonstrated significant differences (*p* < 0.05, cluster level-FWE corrected, Table 3). The increased ReHo value was observed in the Temporal_Inf_L, Cerebelum_6_R, and Hippocampus_R, while decreased ReHo values were observed in the Putamen_L, Insula_R, and Calcarine_R (Figures 4, 5).

**Table 3:** Brain region differences between HC and ID based on ReHo.

Brain regions^a^	Voxel (mm^3^)	AAL	MNI coordinates			*T* value
			*x*	*y*	*z*	
HC < ID						
*Cluster 1*	223		−63	−54	−18	6.0205
Temporal_Inf_L	112					
*Cluster 2*	184		42	−36	−36	5.5462
Cerebelum_6_R	27					
*Cluster 3*	99		39	−18	−15	5.4838
Hippocampus_R	55					
HC > ID						
*Cluster 1*	640		−30	−12	3	−5.5898
Putamen_L	95					
*Cluster 2*	948		42	6	3	−5.404
Insula_R	112					
*Cluster 3*	72		3	−57	12	−4.2716
Calcarine_R	8					

^a^Peak point of the selected brain region cluster.

### 3.4 Scale correlations

Pearson correlation analysis results indicated no significant correlations between ALFF values, ReHo values in the right insula and right central sulcus cap, and Insomnia Severity Index, the HAMD scale scores in men with ID (*p* > 0.05).

## 4 Discussion

In this study, both ALFF and ReHo values were significantly changed in male patients with insomnia compared with HCs, particularly in regions such as the Precentral gyrus of the sensorimotor network, the insula of the salience network, hippocampus of the memory system and the Cerebellar hemispheres, the Temporal lobe, Putamen of the basal ganglia and the Calcarine cortex of the visual system. These findings confirm that individuals suffering from insomnia exhibit neuronal activities changing across multiple brain areas. This widely distributed pattern of abnormal brain activity provides new perspectives on understanding the neural mechanisms of insomnia.

Compared with the HCs, male insomnia patients exhibited increased ALFF in the precentral gyrus. This finding aligns with the study by Wang et al. (2025), which demonstrated altered ALFF values in the precentral gyrus of insomnia patients. As a crucial hub of the sensorimotor network, the elevated ALFF values in this region may reflect an unstable state of the motor control system in insomnia patients. This regional functional hyperactivity could amplify motor intention signals through enhanced neural activity, resulting in characteristic somatic discomfort and motor restlessness during wakefulness. These findings provide neuroimaging evidence supporting the “hyperarousal” hypothesis of insomnia.

The insula serves as a crucial node within the neural network, playing a significant role in self-awareness, subjective salience, and cognitive processes. Numerous studies have demonstrated that the insula is also vital during sleep (Wang et al., 2022). Furthermore, some research has indicated that alterations in the ReHo values of the insula may reflect abnormal changes in emotional circuit function (Wang et al., 2016). Our findings reveal a decrease in insular ReHo values among male patients with insomnia. Additionally, certain studies have indicated that voxel-based morphometry (VBM) results of the insula are negatively correlated with PSQI scores, suggesting that reduced activity in this region may contribute to insomnia (Yin et al., 2021), which is consistent with our findings and implies potential stage-specific functional changes of the insula in the pathological process of insomnia.

In contrast to our study, Wang et al. (2016) reported an increase in insular activation within their rs-fMRI analysis of patients with insomnia. They posited that this finding indicates an abnormal activation state of the insula associated with anxiety and negative emotional states experienced by these individuals. Jiang et al. (2023) further observed that insomnia patients exhibited higher ALFF values in the insula compared to healthy controls, noting an increase in activity correlating with greater severity of insomnia. These discrepancies may reflect different developmental stages or subtypes of insomnia, or methodological differences across studies, pointing toward important directions for future research.

Recently, an increasing number of researchers have begun to incorporate changes in the cerebellum into their research. Previous studies have indicated that the cerebellum is also linked to emotional regulation and cognitive function (Hoche et al., 2017). The alteration of cerebellar function is another particular finding of this study. We observed concurrent increases in both ALFF and ReHo values in the right Cerebellum_6 region, forming an interesting contrast with reports of reduced cerebellar gray matter volume by Jung et al. (2019) and decreased cerebellar activity by Fasiello et al. (2022) and Li C. et al. (2016). In brief, these findings suggest abnormal activity within the cerebellum among patients with insomnia. We hypothesize that this enhanced neural synchronization may reflect differential neuroregulatory mechanisms between sexes.

Neuroimaging evidence demonstrates structural deterioration in the temporal cortex of insomnia patients, characterized by gray matter volume reduction and compromised white matter integrity, that elevates susceptibility to cognitive impairment (Zhang et al., 2024). In our study, there was an increasing trend in ReHo values in both the temporal lobe and hippocampus within this region among male insomniacs. This “elevated functional activity but structural damage” dissociation pattern may reflect neural adaptive changes induced by chronic sleep deprivation. This observation aligns with established sexual dimorphism in temporal lobe memory networks (Dai et al., 2012). The function of the hippocampus, given its central role in declarative memory consolidation (Kumaran and Maguire, 2005), is likely directly related to the memory dysfunction commonly observed in insomnia patients (Abdelhack et al., 2023). This finding provides an important neurobiological basis for understanding insomnia-related cognitive impairment.

The reduction of activity in the Putamen and Calcarine further suggests that it may present characteristic neuroimaging manifestations specific to male patients phenomenon warranting further investigation across larger sample sizes.

Although depressed patients were excluded from this study, mood-related alterations in brain function persisted, indicating that insomnia among male patients remained somewhat associated with negative emotions or psychological factors.

The multimodal approach employed here offers a more comprehensive perspective. However, it is important to note that this study was conducted at a single center, and multicenter investigations could enhance diagnostic efficacy. The relatively small sample size and exclusive inclusion of male participants may somewhat limit the generalizability of the findings. Including female subjects would provide broader representation and insights into gender differences, and in terms of imaging acquisition parameters, the relatively short scan duration and specific TR settings may have certain impacts on the calculation accuracy of ALFF and ReHo metrics. Future studies employing optimized imaging parameters and a higher field MRI scanner would help improve the reliability of measurement results.

## 5 Conclusion

In summary, results derived from rs-fMRI revealed reduced ALFF values in the Precentral_R and increased ALFF values in the Cerebelum_6_R and Temporal_Inf_L. The increased ReHo values were observed in the Temporal_Inf_L, Cerebelum_6_R, and Hippocampus_R, while decreased ReHo values were observed in the Putamen_L, Insula_R, and Calcarine_R, among male patients diagnosed with primary insomnia. Furthermore, abnormal activities within the cerebral regions associated with emotional activity may serve as a distinctive neuroimaging feature for male individuals experiencing primary insomnia. These findings provide important information about the neurobiological mechanisms and neuroimaging features of primary insomnia in men.

## Data Availability

The original contributions presented in the study are included in the article/supplementary material, further inquiries can be directed to the corresponding authors.
